# Patient and Medical Oncologists’ Perspectives on Prescribed Lifestyle Intervention—Experiences of Women with Breast Cancer and Providers

**DOI:** 10.3390/jcm9092815

**Published:** 2020-08-31

**Authors:** Lynda G. Balneaves, Tracy L. O. Truant, Cheri Van Patten, Amy A. Kirkham, Erin Waters, Kristin L. Campbell

**Affiliations:** 1 College of Nursing, University of Manitoba, Winnipeg, MB R3T 2N2, Canada; lynda.balneaves@umanitoba.ca; 2 BC Cancer, Vancouver, BC V5Z 1G1, Canada; ttruant@shaw.ca (T.L.O.T.); cvpatten@bccancer.bc.ca (C.V.P.); 3 Faculty of Kinesiology and Physical Education, University of Toronto, Toronto, ON M5S 2W6, Canada; amy.kirkham@utoronto.ca; 4 School of Nursing, University of British Columbia, Vancouver, BC V6T 2B5, Canada; erinmccarrell@hotmail.com; 5 Department of Physical Therapy, Faculty of Medicine, University of British Columbia, 212, 2177 Wesbrook Mall, Vancouver, BC V6T 1Z3, Canada

**Keywords:** breast neoplasms, diet therapy, exercise, non-drug, prescriptions, qualitative research

## Abstract

This study explored the perspectives and experiences of breast cancer patients and medical oncologists with regards to participation in a lifestyle intervention at a tertiary cancer treatment center. A thematic approach was used to understand the context within which a lifestyle intervention was recommended and experienced, to inform future lifestyle programming and promote uptake. Twelve women with breast cancer receiving adjuvant chemotherapy and eight medical oncologists completed interviews. Findings suggest receiving a prescription for a lifestyle intervention from a trusted health professional was influential to women with breast cancer. The intervention offered physical, psychological, emotional, social, and informational benefits to the women and oncologists perceived both physiological and relational benefit to prescribing the intervention. Challenges focused on program access and tailored interventions. Lifestyle prescriptions are perceived by women with breast cancer to have numerous benefits and may promote lifestyle interventions and build rapport between oncologists and women. Oncology healthcare professionals play a pivotal role in motivating women’s participation in lifestyle interventions during breast cancer treatment. Maintenance programs that transition patients into community settings and provide on-going information and follow-up are needed.

## 1. Introduction

There is compelling evidence that exercise is safe for breast cancer patients during treatment and helps to manage many common side effects, as well as enhance quality of life, and overall health of cancer survivors [1,2]. Furthermore, there are increasing calls for exercise to be adopted as part of standard of care in oncology in Canada [3] and internationally [4]. Cancer patients, however, report unique challenges to exercise adoption and maintenance, including concerns about safety, desire for guidance from exercise professionals trained to work with cancer patients, and physical limitations related to treatment side effects [5,6,7,8].

Research has shown that women with newly diagnosed breast cancer may increase exercise when referred by their oncologist [9]. Cancer patients in North America, however, currently have limited access to exercise programming outside of research protocols, especially during adjuvant treatment when the need is greatest with regards to symptom management and prevention of chemotherapy-associated deconditioning. There exists a gap between the scientific evidence and clinical practice regarding the use of exercise in the management of breast and other cancers. Potential reasons for the limited knowledge translation were well described by Santa Mina and colleagues [10]. These include, among other factors, “an impression among clinicians that exercise may increase the risk of injury, fatigue and exacerbation of symptoms; overwhelmed and financially drained clinical programs; physical space restrictions; overall lack of clinicians with relevant exercise and clinical education and experience; and lack of discussion between patient and physician about exercise” [10] (p. e137).

While there is emerging research regarding the experience of cancer patients involved in lifestyle interventions [11,12,13,14], there is limited research examining patients’ perspectives regarding how lifestyle interventions are introduced and framed as part of comprehensive treatment within a cancer care setting. Specifically, what are patients’ perceptions and experiences when exercise and nutrition programming are prescribed by their oncologists? There has been even less exploration of the experience and perceptions of health professionals who have been engaged in referring patients to such lifestyle programs. One exception is the work of Smaradottir et al., who explored the perception of cancer patients and oncology healthcare professionals regarding the role of exercise in cancer care [15]. Healthcare professionals were concerned about recommending exercise to frail and sedentary cancer patients and most preferred to defer to a specialist [15]. Further understanding oncologists’ perceptions regarding their role in recommending exercise and nutrition interventions, as well as the challenges they face within busy ambulatory care settings, will shape future programming related to lifestyle behaviors in cancer care.

Our research team tested the feasibility of delivering a lifestyle intervention, the Nutrition and Exercise during Adjuvant Treatment (NExT) study, as part of standard of care for early stage breast cancer patients who were scheduled to receive adjuvant chemotherapy [16,17]. Medical oncologists at a cancer center in Vancouver, Canada discussed the study with potentially eligible women. The oncologist completed a “prescription” form in duplicate (i.e., one copy to patient and one to the study staff) recommending the intervention. All referred women were screened. The intervention consisted of a supervised aerobic and resistance training intervention coinciding with adjuvant chemotherapy and radiation treatment (3 times/week for a mean of 25 weeks). This was followed by a transition to increased self-directed exercise and reduced frequency of supervised sessions (twice/week for 10 weeks, then once/week for another 10 weeks) such that the total prescription consisted of 150 min/week of moderate-intensity aerobic exercise and twice weekly resistance exercise. Participants were also invited to participate in a group-based nutrition counselling session, led by a registered dietitian with experience in working with breast cancer patients. Further details of the program, including outcomes, have been reported elsewhere [16,17].

The purpose of the current study, a sub-study of the NExT parent study, was: (1) to explore breast cancer patients’ perspectives and experiences of being prescribed and subsequently engaging in exercise and nutrition programming at a large tertiary cancer treatment center; and (2) to explore medical oncologists’ perspectives and experiences in referring breast cancer patients to this intervention. The primary aim was to better understand the context within which the lifestyle intervention was recommended and experienced to shape future lifestyle programming and promote uptake in cancer care settings.

## 2. Materials and Methods

This sub-study used a qualitative descriptive approach [18]. Eligible patients for the NExT parent study included English-speaking, adult (>18 years) women diagnosed with stage I–IIIA breast cancer, who were receiving adjuvant chemotherapy, and had medical approval and a referral by their medical oncologist to participate in the lifestyle intervention. Potential participants were excluded if they had a BMI of ≥ 40 kg/m^2^, had mobility issues, or unstable cardiovascular disease or diabetes. To participate in the sub-study, women also had to have completed participation in the exercise component of the NExT parent study and were no longer receiving adjuvant chemotherapy. For further information about the NExT parent study, please see Kirkham et al.

Purposive sampling was used to invite patients and the medical oncologists who had participated in the parent study to join the sub-study. Diversity in age, socioeconomic status, geographical dwelling (rural/urban), and ethnicity was sought in the patient sample. All eight oncologists who had participated in the NExT parent study and had provided care to the participants were invited to take part in the sub-study.

Forty-three women who participated in the NExT study and eight oncologists were emailed letters of invitation. Interested women and oncologists were asked to contact the research team to arrange a time to complete a brief telephone or in-person interview. The NExT study and sub-study were approved by the University of British Columbia’s Clinical and Behavioural Research Ethics Boards (H12-02504 and H14-02805) and all procedures performed were in accordance with the 1964 Helsinki Declaration and its later amendments. All participants provided written informed consent. We confirm all patient/personal identifiers have been removed or disguised so the patient/person(s) described are not identifiable and cannot be identified through the details of the story.

### 2.1. Data Collection

One-on-one, semi-structured interviews were conducted with women and oncologists. The breast cancer patients’ interviews focused on their perceptions and experiences of having an oncologist prescribe a lifestyle intervention, their perception of the facilitators and challenges related to uptake and engagement in the intervention, and their recommendations regarding the role of oncologists and other health professionals in promoting lifestyle change. Oncologists’ interviews focused on how they perceived exercise and nutrition as part of conventional breast cancer treatment, their approach to introducing the NExT study to potential subjects, how they determined study eligibility, and their perceptions of the facilitators and challenges experienced by patients regarding study participation. Interviews were digitally recorded and transcribed verbatim, without identifying information. The interviews, which took 30–60 min to complete, were conducted by one team member (EW), who is experienced in qualitative research and a registered nurse. Brief questionnaires assessing patient’s demographics were completed, with treatment and diagnosis characteristics extracted from medical records.

### 2.2. Data Analysis

The interview data were analyzed beginning with line-by-line coding, with keywords, phrases, and concepts identified. Using a thematic analysis approach, this coding was then consolidated into a formal coding scheme following consultation with the research team. The coding scheme was piloted on two interviews (one patient and one oncologist) by two investigators (TT and LB) and any discrepancies or overlap in coding was discussed, with revisions made to the coding scheme. The interviews were then coded by hand using the revised coding scheme. Using this “codebook” approach to thematic analysis [19], data which did not fit the coding scheme resulted in a new code beginning developed. Data were extracted based on the coding and reviewed for major themes and concepts. With regards to rigor, an audit trail was kept of all coding decisions as well as team meetings discussing the developing themes and concepts. A preliminary summary of the study findings was sent to the research team to ensure the trustworthiness of the data. No member checking with participants was undertaken due to time and costs constraints.

## 3. Results

### 3.1. Sample Demographics

Of the 43 women invited to participate in the study, 18 women initially expressed interest in taking part in the research; however, six women were not able to be interviewed due to being out of town or not confirming interview appointments. Those who did not respond to the study invitation (*n* = 25) did not provide a reason for the lack of response. Table 1 describes the demographic and disease characteristics of the 12 women who ultimately participated in the study. The majority were middle-aged (mean of 53.8 years), married (83%), had some post-secondary education, and reported a personal income of ≥CA$50,000 (84%). The majority had stage II breast cancer (67%). Table 2 summarizes the demographic and training characteristics of the eight oncologists who took part in the study.

### 3.2. Perceptions of the “Prescription”

For most women, their oncologist’s expertise and credibility significantly influenced their decision to participate. Tailored and positive statements by the oncologist regarding how the intervention would help women cope with their diagnosis and side effects persuaded several to join the study. However, the perceived value of a “prescription” was mixed. Most oncologists thought that having a physician prescribe a lifestyle intervention was more powerful than a simple suggestion and was taken “more seriously” by patients and their families. In contrast, survivors did not talk about the prescription as a strategy that promoted their uptake. Instead, the way in which oncologists introduced the intervention in a compassionate and respectful manner was perceived to greatly encourage participation. A trusting relationship with one’s oncologist was also perceived as being essential prior to participating in the intervention:

I would respond to somebody that I had a relationship with and who I knew. I would say there has to be a trust relationship before it can be introduced. And then it’s less important—if there’s already a relationship—it’s less important how it’s introduced, and more important that you trust the person.

Perspectives on which healthcare professional should introduce the lifestyle intervention were mixed. Some women felt that an oncologists’ recommendation reinforced the safety and efficacy of the intervention. Several patients and oncologists, however, suggested nurses be “front and center” in providing lifestyle recommendations. An overarching sentiment expressed by both women and oncologists was the need for all healthcare professionals to become more knowledgeable about nutrition and exercise, and to consistently address lifestyle as a core part of cancer treatment and care. As one oncologist shared: “I feel very strongly that exercise should be a part of our patients’ recovery protocol.”

### 3.3. Benefits of the Lifestyle Intervention

#### 3.3.1. Reframing Disease as Health

My mother is a breast cancer survivor, and she stayed on the couch. I was a breast cancer survivor, and I hauled myself to the gym. I went hiking during my cancer treatment. I actually embraced life and lived during my treatment.

An overarching concept that arose when discussing the benefits of the intervention with patients and oncologists was that it encouraged the reframing of disease as health. The intervention helped women move beyond “… being the victim, being the patient, and just telling your sob story”. Rather than focusing on what they could not do because of cancer, the women were empowered to focus on what they could do. For others, their ability to push through “bad days” promoted feelings of control over their health:

What’s taken away from you is your health through the actual treatment. I had some control over that, I could exercise through it. You know … not feeling great at all, but not just getting through the day, but actually exercising is psychologically strong!

The intervention also helped some women shift their self-image and move beyond their illness: “I think that in any other situation, you feel like a patient. But there [in the gym] I almost felt like an athlete. It wasn’t about being sick. It was about improving your fitness level.”

Several oncologists also spoke of how discussing the lifestyle intervention allowed them to reframe the conversation from being negatively focused on disease and treatment to instead encouraging women to regain their health and well-being. This shift had the welcome outcome, for some oncologists, of improving their rapport with women by giving them a chance to offer a therapy that was overwhelmingly beneficial and associated with minimal risks.

It was easy to offer and recruit—when patients are feeling very overwhelmed and losing control because of cancer, then having something that they can do proactively—that in itself is therapeutic.

#### 3.3.2. Coping with the Physical Consequences of Cancer and Beyond

Many of the oncologists spoke of the “compelling evidence” that lifestyle interventions support individuals on adjuvant cancer treatment by decreasing side effects and improving overall fitness. Women, despite feeling unwell, described how the intervention increased their physical activity, helped them lose weight, managed their side effects, and allowed them to “take better care of my body”. The women reported that they hoped the intervention would create a habit that they could maintain into survivorship.

Women and oncologists remarked on the supportive nature of the intervention in promoting adherence to exercise. Of note, attendance averaged 60% (SD 26%), 52% (SD 33%), and 50% (SD 38%) for thrice, twice, and once weekly sessions, respectively, throughout the study.

#### 3.3.3. Managing Distress

Women perceived the psychological benefits of the lifestyle intervention to outweigh the physical benefits, with the intervention playing a crucial role in managing their distress and the “terrible thoughts and worries” that accompanied a cancer diagnosis. As one woman described, “the gym was an incredible tonic … and it immediately improved my mood. At the end of every session I would feel better than when I arrived.” Other women described the lifestyle intervention as “cheaper than therapy”, offering ongoing psychosocial support.

The doctor’s going to only do so much, and they deal with the medication and the surgery and the following you. Obviously, you need that to survive. But if you’re going to start building up some strength again and energy and confidence and then the emotional support that I got from being at the gym, where are you going to get that? Clearly the body, I think, recovers better when it’s being looked after in every way.

For some women, the intervention offered a sense of control and motivation to “get out of the house” and a way to “order my days”, especially for those not working because of their illness.

#### 3.3.4. Gaining Social Support

The group-based intervention and exclusivity of the gym created a “healthy sense of community” and safe environment for women to share their concerns and gain information about cancer, exercise, and nutrition. For many, the supportive network maintained its bonds even after formal participation in the intervention ended:

One person, she said, ‘We go to tea on this day. Do you want to join us?’ and we developed a posse. We also started a book club, we try to hike once a month, we try to get together once a while for a dinner. And if one person has a question … she emailed her posse and you would not believe the fan-out of response from other people with the same situation.

Several women reflected on the unique support offered by other participants and how it differed from that provided by their social network. The intervention provided them with a safe place to work through the challenges that come with cancer and treatment without burdening their family.

I didn’t give too much information to my kids or family … just the basics because I didn’t want him to stress out because he’s stressed already, worrying about me. So, a lot of it was with the ladies at the gym. They really provided a lot of support because we could laugh about it, and they would know what we’re going through. You need people who will listen and understand what you’re going through. And then you deal with it and move forward.

An additional benefit of the intervention was that social expectations were created among the women that they would show up for scheduled sessions, despite feeling unwell. The supportive and motivational culture of the NExT study was also noted by the oncologists: “There was this camaraderie element that developed among participants because they were all in the same boat at the same time, probably also helped motivation.”

#### 3.3.5. Perceived Challenges of the Lifestyle Intervention

Several women experienced substantial difficulty in regularly attending the program. Beyond treatment side effects, several patients reported that life responsibilities, such as childcare and employment, were important barriers. The restricted gym hours (i.e., 9 a.m. to 3 p.m.) contributed to challenges with attendance, especially upon treatment completion when patients resumed regular activities. The location of the gym near the province’s largest cancer treatment center was problematic for some women who lived at a distance and prevented some from participating due to the travel required.

Despite many patients praising the supportive aspects of the intervention, a few were disappointed with how it was tailored. For example, one younger woman did not feel that her unique needs were acknowledged with the exercise prescription, which she perceived to be designed for older women and unable to account for prior exercise experience. The program was structured to begin with moderate-intensity exercise concurrent with starting chemotherapy, and, despite being provided with an advanced exercise prescription for more experienced participants [20], she felt dissuaded from performing high-intensity exercise.

Although most patients found the social aspect of the lifestyle intervention to be supportive and affirming, at least one woman reflected that there might be individuals for whom this aspect of the program may be problematic:

It can be a bit challenging for some people. A lot of people talking about cancer and chemo, it can get a bit depressing. Some people like to talk about it, others don’t really like to talk about it. So, there is also that emotional, psychological aspect of it that you want to be careful with, because it really depends on who they are and how they are living with their situation.

Oncologists perceived few problems associated with the intervention. One physician, however, did caution that, while prescribing the intervention may be motivating for many patients, it may cause guilt in those not following through with the recommendation. Some oncologists also expressed concerns that older individuals who lacked a basic level of fitness or had pre-existing physical disabilities (e.g., joint replacement surgery) may require a modified exercise prescription to engage in the intervention. As one oncologist shared: “… there is a population of older woman who cannot exercise quite as intensely, maybe because they have joint issues or just that many more accumulated years of lack of fitness.”

### 3.4. Participant Recommendations

Oncologists and most patients believed the intervention should be offered as a standard part of breast cancer care, and as early as possible within the cancer trajectory, before the onset of treatment side effects could limit individuals’ enthusiasm and/or perceived capacity to participate. The women and oncologists recommended a number of modifications to the content and structure of the lifestyle intervention program (see Table 3).

## 4. Discussion

The study findings offer important insights into the facilitators and challenges of prescribing a lifestyle intervention for women with breast cancer undergoing adjuvant chemotherapy. While medical oncologists perceived a prescription as a significant motivating factor for breast cancer patients, the women described being motivated by the potential benefits of attending a breast cancer-specific lifestyle intervention, and their desire to improve their well-being following diagnosis. Which specific healthcare professional provided the prescription appeared to be less important to patients, other than it being a clinician with whom they had a trusting relationship. These findings were similar to Park et al., who found that an exercise recommendation solely from an oncologist was insufficient in motivating general exercise uptake in a breast and colorectal cancer population [21]. Recommendations that were accompanied with an exercise motivation package (i.e., exercise DVDs, pedometer, diary, and education), however, were significantly more likely to result in increased exercise frequency and intensity [21]. In contrast, Befort et al. found oncologist referrals to a lifestyle intervention for rural breast cancer survivors resulted in the highest enrolment rate [22]. Taken together, these findings suggest that with the proper support, a cancer diagnosis may be a teachable moment for breast cancer patients to engage in risk-reducing health behaviors that hold the potential to improve morbidity and mortality [23].

The benefits gained by patients went well beyond symptom management and improved physical fitness during adjuvant treatment. The program offered a trusted community where women’s psychosocial and physical needs could be addressed in a supportive manner. There has been increasing attention on the potential efficacy of non-traditional support groups, such as dragon boat teams [24] and men’s sheds [25], in addressing complex physical and psychosocial needs in a range of patient groups. The success of such groups in promoting healthy lifestyle behavior is, in part, a consequence of the sense of community, camaraderie, and purpose that develops among members.

The challenges reported by patients and oncologists focused mainly on contextualizing the lifestyle intervention to the unique needs of individuals (e.g., age, fitness goals, work, and family commitments). The lifestyle program used evidence-based guidelines to tailor the intervention to participants’ needs; however, social determinants of health or other factors that may have impacted individuals’ uptake of the intervention [26,27] were not addressed. Buffart et al. [28], in their summary of the current knowledge gaps in physical activity for cancer survivors, described the importance of moving beyond a one-size-fits all approach and tailoring exercise guidelines for individual characteristics, contexts, and capabilities for optimal uptake.

The findings have key practice implications. Oncologists and nurses can play a pivotal role in highlighting the importance and safety of lifestyle interventions during breast cancer treatment, and in motivating participation through tailored recommendations, and referrals to other health professionals, such as registered dietitians, physiotherapists, and registered kinesiologists, or to established community programming. Engaging primary care providers, such as family physicians and nurse practitioners, and ensuring nutrition and exercise are included in survivorship care plans will promote long-term support for cancer patients’ and survivors’ lifestyle modification maintenance. Continuing education, however, will be required to inform health professionals about the benefits of lifestyle interventions following a cancer diagnosis and to dispel myths regarding the ability of patients to engage in lifestyle modification while undergoing adjuvant cancer therapy. Recent Canadian and American surveys demonstrate that oncology nurses see the value of lifestyle interventions, and perceive patients to be receptive, but lack the necessary knowledge and counseling skills [29,30].

Acknowledging the time and fiscal constraints in ambulatory cancer care settings, a streamlined process is required to support oncologists, nurses and other health professionals in prescribing and referring patients to lifestyle interventions. Utilizing peer mentors and volunteers in recruiting patients and supporting adherence may facilitate uptake of the lifestyle program with minimal impact on healthcare staff and resources. Accessibility may be enhanced through e-health technologies. Recent demonstrated efficacy of online mindfulness meditation programs [31] and support groups [32] reinforce e-technology use in promoting well-being among cancer patients. In addition, embedding the lifestyle intervention within regional oncology programs as well as local community centers with more flexible hours and locations may improve adherence, especially among younger patients with work and family responsibilities.

The study findings are not transferable to all lifestyle interventions situated in cancer care settings given the urban setting, small sample size, the use of an exercise and nutrition programming “prescription”, and the focus on breast cancer. In addition, the sample was primarily comprised of white or Asian, well-educated, and affluent women. The perspectives of those who did not participate were not captured, which may offer unique insights into the challenges of prescribing nutrition and exercise to newly diagnosed cancer patients.

## 5. Conclusions

Our findings support the need for exercise and nutrition to be embedded in standard cancer care and acknowledged early, and throughout, the breast cancer trajectory to address not only important physical and psychosocial needs experienced by patients undergoing adjuvant cancer treatment [33,34,35], but also potentially improve all-cause mortality and reduce risk of recurrence [36]. Including rehabilitative programs as a standard part of care may further reduce the economic burden experienced by women with breast cancer experiencing long-term adverse effects [37]. Despite these important outcomes, implementing sustainable lifestyle programs in the clinical setting remains a challenge in cancer care. While high-quality exercise guidelines are available, other barriers must be addressed, such as space restrictions in cancer centers and out-of-pocket costs and travel time for patients [10,38].

Although lifestyle prescriptions may be an efficient and familiar way for healthcare professionals to promote exercise and nutrition in cancer care settings, it is not as powerful a motivator as the content itself and the perceived physical, emotional, and social benefits. Lifestyle interventions have the potential to improve health outcomes, as well as provide women with a unique peer community for support and empowerment, and an opportunity to reframe their illness in a positive manner.

The study findings clearly indicate that cancer patients require support during but also after completion of treatment to adopt healthy lifestyle habits. Maintenance programs that transition patients into community settings and provide them with on-going information and follow-up about nutrition and exercise will reinforce the knowledge and skills gained during an initial lifestyle program that takes place during treatment in a clinical setting. Further, such maintenance programs have been found to support continued problem solving, empower patients to become more self-directed, and actively bolster self-accountability and motivation [39]. Linking to existing community programs (i.e., cardiac rehabilitation) may provide a setting in which cancer patients and survivors can network and support one another [40].

## Figures and Tables

**Table 1:** Breast cancer survivor demographic and disease characteristics (*n* = 12).

Characteristics	*n* (%)
*Age* (years)	Mean 51.9 ± 14.2 SD (Range 30–72)
*Marital status*	
Married/Common-law	10 (83.0)
Single	1 (8.0)
Widowed	1 (8.0)
*Education*	
Some university	3 (25.0)
Bachelor’s degree	3 (25.0)
Above a bachelor’s degree	6 (50.0)
*Personal income*	
<$50,000	2 (16.0)
$50,000–79,999	3 (25.0)
$80,000–125,000	5 (42.0)
Prefer not to answer	1 (8.0)
Missing	1 (8.0)
*Ethnicity*	
Caucasian	8 (67.0)
Asian	3 (25.0)
African Canadian	1 (8.0)
*Pre-diagnosis work status*	
Full time	6 (50.0)
Self-employed	3 (25.0)
Retired	2 (17.0)
Full time student	1 (8.0)
*Work status during treatment*	
Working	2 (17.0)
Working part-time/casual	2 (17.0)
Not working	8 (67.0)
*Menopausal status*	
Premenopausal	4 (33.0)
Postmenopausal	7 (58.0)
Perimenopausal	1 (8.0)
*Cancer diagnosis*	
Stage I	4 (33.0)
Stage II	8 (67.0)

**Table 2:** Demographic and training characteristics of oncologists (*n* = 8).

Characteristics	*n* (%)
*Gender*	
Female	6 (75.0)
Male	2 (25.0)
*Age*	
30–39	2 (25.0)
40–49	2 (25.0)
50–59	3 (37.5)
Missing	1 (12.5)
*Training*	
Medical oncologist	7 (87.5)
General Practitioner in Oncology	1 (12.5)
*Years of Practice*	
5–9 years	2 (25.0)
10–14 years	2 (25.0)
≥15 years	4 (50.0)

**Table 3:** Recommended Modifications to the Lifestyle Intervention Program.

Program Characteristics	Recommended Modifications
Awareness	Introduce lifestyle program before adjuvant cancer treatment and offer throughout cancer trajectory *^,^^+^ Include lifestyle program brochure in new patient package * Expand lifestyle “prescription” to other healthcare professionals to encourage participation * Dissemination of vignettes from program participants to new patients ^+^ Brief education session for patients and family members on role of exercise and diet during cancer treatment *^,^^+^ Posters in clinical areas encouraging patients to ask health care professionals about lifestyle behaviors *^,^^+^ Include information about diet and exercise into consults about treatment protocols and management of side effects *^,^^+^ Peer mentors (i.e., former program participants) ^+^
Structure	Integration of exercise and diet coaching (i.e., dietitians at the gym) ^+^ Assessment of emotional wellbeing throughout program, with referrals to psychosocial support, as necessary ^+^ Offer lifestyle program in other regional centers and communities as a standard part of breast cancer care *^,^^+^ Develop long-term lifestyle networks in community to provide long-term support to survivors *^,^^+^ Develop alternatives to address accessibility issues for patients related to location and hours of operation (i.e., community-based programs) *^,^^+^ Offer program to other cancer types, but maintain disease-specific groups to promote peer support and social support ^+^ Consider developing age-related cohorts to allow patients at similar life stages to connect *^,^^+^ Ensure environment minimizes risk (e.g., infection) and promotes physical (e.g., supervision by exercise professionals knowledgeable about cancer) and psychological (e.g., remove wigs without feeling self-conscious) safety ^+^
Content	Supplementary print material on exercise and diet with links to credible information resources *,+ Tailor lifestyle program to women’s individual diet and exercise history prior to cancer diagnosis *,^+^ Maintain positive and encouraging approach of trainers ^+^ Tailor lifestyle program to needs of older women living with possible physical limitations (e.g., joint replacements) * Offer program in languages other than English (i.e., printed material and multi-lingual trainers and volunteers *

^+^ Recommended by women with breast cancer. * Recommended by oncologists.

## References

[1] Patel A.V. (2019). American College of Sports Medicine Roundtable Report on Physical Activity, Sedentary Behavior, and Cancer Prevention and Control. Med. Sci. Sports Exerc..

[2] Campbell K.L., Winters-Stone K.M., Wiskemann J., May A.M., Schwartz A.L., Courneya K.S., Zucker D.S., Matthews C.E., Ligibel J.A., Gerber L.H. (2019). Exercise Guidelines for Cancer Survivors: Consensus Statement from International Multidisciplinary Roundtable. Med. Sci. Sports Exerc..

[3] Segal R., Zwaal C., Green E., Tomasone J.R., Loblaw A., Petrella T. (2017). Exercise for People with Cancer Guideline Development, G. Exercise for people with cancer: A clinical practice guideline. Curr. Oncol..

[4] Schmitz K.H., Campbell A.M., Stuiver M.M., Pinto B.M., Schwartz A.L., Morris G.S., Ligibel J.A., Cheville A., Galvao D.A., Alfano C.M. (2019). Exercise is medicine in oncology: Engaging clinicians to help patients move through cancer. CA Cancer J. Clin..

[5] Ottenbacher A.J., Day R.S., Taylor W.C., Sharma S.V., Sloane R., Snyder D.C., Kraus W.E., Demark-Wahnefried W. (2011). Exercise among breast and prostate cancer survivors--what are their barriers?. J. Cancer Surviv. Res. Pract..

[6] Rogers L.Q., Matevey C., Hopkins-Price P., Shah P., Dunnington G., Courneya K.S. (2004). Exploring social cognitive theory constructs for promoting exercise among breast cancer patients. Cancer Nurs..

[7] Sander A.P., Wilson J., Izzo N., Mountford S.A., Hayes K.W. (2012). Factors That Affect Decisions About Physical Activity and Exercise in Survivors of Breast Cancer: A Qualitative Study. Phys. Ther..

[8] Whitehead S., Lavelle K. (2009). Older breast cancer survivors’ views and preferences for physical activity. Qual. Health Res..

[9] Jones L.W., Courneya K.S., Fairey A.S., Mackey J.R. (2004). Effects of an oncologist’s recommendation to exercise on self-reported exercise behavior in newly diagnosed breast cancer survivors: A single-blind, randomized controlled trial. Ann. Behav. Med..

[10] Mina D.S., Alibhai S.M., Matthew A.G., Guglietti C.L., Steele J., Trachtenberg J., Ritvo P.G. (2012). Exercise in clinical cancer care: A call to action and program development description. Curr. Oncol..

[11] Demark-Wahnefried W., Rogers L.Q., Alfano C.M., Thomson C.A., Courneya K.S., Meyerhardt J.A., Stout N.L., Kvale E., Ganzer H., Ligibel J.A. (2015). Practical clinical interventions for diet, physical activity, and weight control in cancer survivors. CA Cancer J. Clin..

[12] Balneaves L.G., Van Patten C., Truant T.L., Kelly M.T., Neil S.E., Campbell K.L. (2014). Breast cancer survivors’ perspectives on a weight loss and physical activity lifestyle intervention. Support. Care Cancer.

[13] Midtgaard J., Hammer N.M., Andersen C., Larsen A., Bruun D.M., Jarden M. (2015). Cancer survivors’ experience of exercise-based cancer rehabilitation—A meta-synthesis of qualitative research. Acta Oncol..

[14] Livsey L., Lewis K. (2018). Breast cancer survivors’ perceptions of participating in a supervised exercise intervention: An exploratory review of the literature. Women Health.

[15] Smaradottir A., Smith A.L., Borgert A.J., Oettel K.R. (2017). Are We on the Same Page? Patient and Provider Perceptions About Exercise in Cancer Care: A Focus Group Study. J. Natl. Compr. Cancer Netw..

[16] Kirkham A.A., Bonsignore A., Bland K.A., McKenzie D.C., Gelmon K.A., Van Patten C.L., Campbell K.L. (2018). Exercise Prescription and Adherence for Breast Cancer: One Size Does Not FITT All. Med. Sci. Sports Exerc..

[17] Kirkham A.A., van Patten C., Gelmon K.A., McKenzie D.C., Bonsignore A., Bland K.A., Campbell K.L. (2017). Effectiveness of oncologist-referred exercise and healthy eating programming as a part of supportive adjuvant care for early breast cancer. Oncologist.

[18] Sandelowski M. (2000). Whatever happened to qualitative description?. Res. Nurs. Health.

[19] Braun V., Clarke V. (2019). Reflecting on reflexive thematic analysis. Qual. Res. Sport Exerc. Health.

[20] Bland K.A., Kirkham A.A., Bovard J., Lloyd M., Shenkier T., Davis M.K., Zucker D., Claydon V.E., Gelmon K.A., McKenzie D.C. (2017). “Chemotherapy-periodized” aerobic exercise for women with breast cancer: A novel exercise prescription to account for fluctuations in fatigue. Appl. Physiol. Nutr. Metab..

[21] Park J.H., Lee J., Oh M., Park H., Chae J., Kim D.I., Lee M.K., Yoon Y.J., Lee C.W., Park S. (2015). The effect of oncologists’ exercise recommendations on the level of exercise and quality of life in survivors of breast and colorectal cancer: A randomized controlled trial. Cancer.

[22] Befort C.A., Bennett L., Christifano D., Klemp J.R., Krebill H. (2015). Effective recruitment of rural breast cancer survivors into a lifestyle intervention. Psychooncology.

[23] Ligibel J.A., Alfano C.M., Courneya K.S., Demark-Wahnefried W., Burger R.A., Chlebowski R.T., Fabian C.J., Gucalp A., Hershman D.L., Hudson M.M. (2014). American Society of Clinical Oncology Position Statement on Obesity and Cancer. J. Clin. Oncol..

[24] Ray H.A., Verhoef M.J. (2013). Dragon boat racing and health-related quality of life of breast cancer survivors: A mixed methods evaluation. BMC Complement. Altern. Med..

[25] Mackenzie C.S., Roger K., Robertson S., Oliffe J.L., Nurmi M.A., Urquhart J. (2017). Counter and Complicit Masculine Discourse Among Men’s Shed Members. Am. J. Men Health.

[26] Alvaro C., Jackson L.A., Kirk S., McHugh T.L., Hughes J., Chircop A., Lyons R.F. (2011). Moving Canadian governmental policies beyond a focus on individual lifestyle: Some insights from complexity and critical theories. Health Promot. Int..

[27] Van der Weijden T., Legare F., Boivin A., Burgers J.S., van Veenendaal H., Stiggelbout A.M., Faber M., Elwyn G. (2010). How to integrate individual patient values and preferences in clinical practice guidelines? A research protocol. Implement. Sci..

[28] Buffart L.M., Galvao D.A., Brug J., Chinapaw M.J., Newton R.U. (2014). Evidence-based physical activity guidelines for cancer survivors: Current guidelines, knowledge gaps and future research directions. Cancer Treat. Rev..

[29] Karvinen K., Bruner B., Truant T. (2015). The Teachable Moment After Cancer Diagnosis: Perceptions from Oncology Nurses. Oncol. Nurs. Forum.

[30] Karvinen K.H., Bruner B., Truant T. (2015). Lifestyle Counseling Practices of Oncology Nurses in the United States and Canada. Clin. J. Oncol. Nurs..

[31] Zernicke K.A., Campbell T.S., Speca M., McCabe-Ruff K., Flowers S., Dirkse D.A., Carlson L.E. (2013). The eCALM Trial-eTherapy for cancer appLying mindfulness: Online mindfulness-based cancer recovery program for underserved individuals living with cancer in Alberta: Protocol development for a randomized wait-list controlled clinical trial. BMC Complement. Altern. Med..

[32] Stephen J., Rojubally A., Macgregor K., McLeod D., Speca M., Taylor-Brown J., Fergus K., Collie K., Turner J., Sellick S. (2013). Evaluation of CancerChatCanada: A program of online support for Canadians affected by cancer. Curr. Oncol..

[33] McNeely M.L., Campbell K.L., Rowe B.H., Klassen T.P., Mackey J.R., Courneya K.S. (2006). Effects of exercise on breast cancer patients and survivors: A systematic review and meta-analysis. Can. Med. Assoc. J..

[34] Mishra S.I., Scherer R.W., Snyder C., Geigle P.M., Berlanstein D.R., Topaloglu O. (2012). Exercise interventions on health-related quality of life for people with cancer during active treatment. Cochrane Database Syst. Rev..

[35] Furmaniak A.C., Menig M., Markes M.H. (2016). Exercise for women receiving adjuvant therapy for breast cancer. Cochrane Database Syst. Rev..

[36] Chen X., Lu W., Zheng W., Gu K., Matthews C.E., Chen Z., Zheng Y., Shu X.O. (2011). Exercise after diagnosis of breast cancer in association with survival. Cancer Prev. Res..

[37] Schmitz K.H., DiSipio T., Gordon L.G., Hayes S.C. (2015). Adverse breast cancer treatment effects: The economic case for making rehabilitative programs standard of care. Support. Care Cancer.

[38] Dennett A.M., Peiris C.L., Shields N., Morgan D., Taylor N.F. (2017). Exercise therapy in oncology rehabilitation in Australia: A mixed-methods study. Asia Pac. J. Clin. Oncol..

[39] Fjeldsoe B.S., Goode A.D., Phongsavan P., Bauman A., Maher G., Winkler E., Eakin E.G. (2016). Evaluating the Maintenance of Lifestyle Changes in a Randomized Controlled Trial of the ‘Get Healthy, Stay Healthy’ Program. JMIR Mhealth Uhealth.

[40] Dolan L.B., Barry D., Petrella T., Davey L., Minnes A., Yantzi A., Marzolini S., Oh P. (2018). The Cardiac Rehabilitation Model Improves Fitness, Quality of Life, and Depression in Breast Cancer Survivors. J. Cardiopulm. Rehabil. Prev..

